# Reliability of Internet- Versus Telephone-Administered Questionnaires in a Diverse Sample of Smokers

**DOI:** 10.2196/jmir.987

**Published:** 2008-03-26

**Authors:** Amanda L Graham, George D Papandonatos

**Affiliations:** ^2^Center for Statistical SciencesBrown UniversityProvidenceRIUSA; ^1^Department of OncologyGeorgetown University Medical Center / Cancer Control ProgramLombardi Comprehensive Cancer CenterWashingtonDCUSA

**Keywords:** Reliability, smoking, Internet, diversity, measurement, psychometrics, minority groups, questionnaires, socioeconomic factors, social class, poverty, African Americans, Hispanic Americans

## Abstract

**Background:**

Smoking is more prevalent among lower-income individuals and certain racial/ethnic minorities. Addressing tobacco cessation among diverse populations is an urgent public health priority. As Internet use continues to rise among all segments of the US population, Web-based interventions have enormous potential to reach priority populations. Conducting Web-based smoking cessation research in priority populations requires psychometrically sound measurement instruments. To date, only one published study has examined the psychometric properties of Internet-administered measures commonly used in Web-based cessation trials. However, the sample was homogeneous with regard to race/ethnicity and income. We sought to replicate and extend these findings in a more diverse sample of smokers.

**Objective:**

The aim was to examine the internal consistency and test-retest reliability of measures commonly used in smoking cessation clinical trials among racial/ethnic minorities and smokers with lower income.

**Methods:**

Participants were enrolled in a randomized trial of the efficacy of an Internet smoking cessation program between June 2005 and September 2006. Following a baseline telephone assessment and randomization into the parent trial, participants were recruited to the reliability substudy. In phase I of recruitment, all participants in the parent trial were recruited to the substudy; in phase II, all consecutive racial/ethnic minority participants in the parent trial were recruited. Race and ethnicity were assessed via self-report using two standard items from the US Office of Management and Budget. An email was sent 2 days after the telephone assessment with a link to the Internet survey. Measures examined were quit methods, perceived stress, depression, social support, smoking temptations, alcohol use, perceived health status, and income. Internal consistency and test-retest reliability of Internet- versus telephone-administered measures were examined within four strata defined by race/ethnicity (non-Hispanic White, racial/ethnic minority) and annual household income (US $40,000 or less, more than $40,000).

**Results:**

Of the 442 individuals invited, 319 participated (72% response rate): 52.4% were non-Hispanic White, 22.9% Black, 11.6% Hispanic, 7.8% Asian, 4.4% American Indian / Alaska Native, and 1% Native Hawaiian / Other Pacific Islander. About half (49.4%) reported an annual household income of US $40,000 or less, and 25.7% had a high school degree or less. Test-retest reliability was satisfactory to excellent across all strata for the majority of measures examined: 9 of 12 continuous variables had intraclass correlation coefficients ≥ 0.70, and 10 of 18 binary variables and both ordinal variables had kappa coefficients ≥ 0.70. Test-retest reliability of several quit methods varied across strata.

**Conclusions:**

Race/ethnicity and income do not affect the psychometric properties of most Internet-administered measures examined. This knowledge adds to the confidence of conducting Web-based smoking cessation research and strengthens the scientific rigor of collecting information via the Internet on racial/ethnic minority and low-income subgroups.

**Trial registration:**

clinicaltrials.gov NCT00282009 (parent trial)

## Introduction

Although the overall prevalence of smoking has declined in recent years, now at 20.9% among US adults, smoking continues to be more prevalent among individuals with lower levels of income and education and among certain subgroups of racial/ethnic minorities [1]. For instance, smoking prevalence is 29.9% among those living below the poverty line, 43.2% among adults with a General Educational Development (GED) diploma, 32.6% among those with 9-11 years of education, 26.7% among African American men, and 32% among American Indians / Alaska Natives [1]. In addition, low education and income also have been linked to lower rates of quit attempts and quit success [2,3]. Given the enormous health burden and economic impact of smoking [4,5], addressing tobacco cessation among diverse populations has been identified as an urgent public health priority [6].

Increasingly, the Internet is being recognized as having great potential to address disparities in health and health risk behaviors (such as tobacco use) by providing information, treatment, and support to traditionally underserved populations [7-12]. More than 70% of US adults now use the Internet [13], and online usage has increased steadily since 2000 across race, education, income, age, and rural/urban categories [14,15]. In 2005, a majority of African Americans (57%) and Latinos (70%) reported using the Internet, as did 49% of individuals living in households with an annual income of less than US $30,000 [14]. In addition to the reach of the Internet, its 24/7 availability, the ability to engage with others as anonymously as desired, and the use of audio, video, and numerous other interactive features make it an appealing dissemination channel for health information and behavior change interventions. Indeed, with thousands of health-related websites in existence, the Internet now plays a meaningful role in the health care system, often serving as the primary source of health-related information and support for consumers.

The use of the Internet among smokers has increased steadily in recent years as well. In 2006, 9% of online adults (more than 10 million people) had searched the Internet for help in quitting smoking [16], up from 6% in 2002 [17]. Studies of Web-based cessation programs are growing rapidly in number [18], with early studies describing the development, usability, and pilot testing of programs [19-24] and more recent reports describing randomized efficacy trials [25-32]. To date, the majority of these studies have focused on “mainstream” Internet users who are largely non-Hispanic White, college educated, and have higher incomes. However, given the growth of Internet use across all demographic subgroups and the recent national attention on eliminating health disparities [33-37], research and development efforts will need to increasingly focus on tobacco use among priority populations such as racial/ethnic minorities [38] and those with lower levels of income and education.

Despite the overall increase in Internet use, it must be acknowledged that access to Web-based cessation programs is still uneven across populations with regard to income and race/ethnicity, with the poor and racial/ethnic minorities having more limited access. However, the persistence of a “digital divide” does not negate the need to conduct rigorous efficacy and effectiveness studies in these subgroups. Rather, it underscores the importance of research to understand for whom, why, and under what conditions Internet cessation programs are effective and to elucidate new directions to further reduce the digital divide.

Critical to the conduct of Web-based cessation research with more diverse populations will be the availability of measurement instruments that have been validated using samples of the target audience [39-41]. The assumption of universal applicability of standardized scales normed on majority populations needs to be explicitly tested across domains (such as racial/ethnic background, income, and education) to ensure that their use with specific subgroups is relevant and appropriate [41]. A growing body of evidence suggests that the reliability and validity of data obtained using questionnaires administered via the Internet are generally consistent with results obtained through paper-and-pencil and computer-administered questionnaires. However, the majority of these studies employ between-group comparisons. Cross-method consistencies examined within subjects have been demonstrated for several constructs, including dietary intake [42], independent life skills among youth [43], health status and health behaviors [44], and psychopathology screening [45].

To date, only one published study that we know of has examined the cross-method consistency of Internet- and telephone-administered measures commonly used in smoking cessation clinical trials using a within-subject design [46]. Our research group found that the internal consistency and test-retest reliability coefficients were comparable for Internet- and telephone-administered measures of stress, depression, self-efficacy, social support, perceived health status, alcohol use, and previous quit methods [46]. However, the sample in this ongoing study was primarily non-Hispanic White (80%) with a household income above US $30,000 (73%). It is important not only to determine that assessment instruments perform adequately when administered via the Internet, but also that they demonstrate sound psychometric properties across subgroups when administered via the Internet [47,48]. Therefore, the goal of the present study was to replicate and extend these findings in a more diverse sample of smokers. Specifically, we were interested in determining whether the psychometric properties of the measures previously examined were comparable across categories of race/ethnicity and income when administered online and by telephone.

## Methods

### Sample Recruitment

Participants were enrolled in a parent study that is an ongoing randomized controlled trial of the efficacy of an Internet smoking cessation program (QuitNet) and telephone counseling (Clinicaltrials.gov: NCT00282009). Recruitment into the parent trial has been described elsewhere [28]. Following a baseline telephone assessment, participants were randomized to treatment and invited to participate in a reliability substudy. Those who agreed were emailed 2 days later with a link to the online survey. Each participant’s unique study identification number was embedded into the link to the online survey so that responses could be joined with their telephone survey data. A description of the online survey administration is available in Graham et al [46]. Participants were paid US $15 for completing the online survey.

Recruitment to the substudy was conducted in two phases. In phase I (June to September 2005), all individuals randomized to the parent trial were recruited. This yielded a sample that was primarily non-Hispanic White with a household income above US $30,000. To increase the heterogeneity of the sample, all racial/ethnic minority participants consecutively randomized to the parent trail were recruited in phase II (October 2005 to September 2006). Race and ethnicity were assessed using the US Office of Management and Budget [49] 2-question format. Participants were first asked to indicate their race from one of five categories: (1) American Indian or Alaska Native: a person having origins in any of the original peoples of North and South America (including Central America), and who maintains tribal affiliation or community attachment; (2) Asian: a person having origins in any of the original peoples of the Far East, Southeast Asia, or the Indian subcontinent including, for example, Cambodia, China, India, Japan, Korea, Malaysia, Pakistan, the Philippine Islands, Thailand, and Vietnam; (3) Black or African American: a person having origins in any of the black racial groups of Africa; (4) Native Hawaiian or Other Pacific Islander: a person having origins in any of the original peoples of Hawaii, Guam, Samoa, or other Pacific Islands; and (5) White: a person having origins in any of the original peoples of Europe, the Middle East, or North Africa. Next, participants were asked to indicate if they were Hispanic or Latino, meaning a person of Cuban, Mexican, Puerto Rican, South or Central American, or other Spanish culture or origin, regardless of race. These categories are required in all federally funded research studies in the United States. The study received human subject protections approval from the Georgetown University Medical Center institutional review board.

### Measures

In the parent trial, the baseline telephone assessment included measures of demographic, smoking, and psychosocial characteristics. To be sensitive to response burden on participants in an Internet-based trial, brief measures and items from large national epidemiologic surveys with known psychometric properties were selected. The present study examined the reliability of the following subset of measures administered via the Internet.

#### Smoking Temptations Questionnaire (Short-Form)

The short-form (9-item) version of the Smoking Temptations Questionnaire [50] assessed the temptation to smoke in different situations. Each item is rated on a 5-point scale ranging from 1 “not at all tempting” to 5 “extremely tempting.” The questionnaire can be scored to form a total score, as well as three subscale scores that measure temptations in positive affect or social situations, negative affect situations, and habitual or craving situations. This short form is derived from a 17-item measure for which internal consistency coefficients are as follows: Positive Affect / Social (6 items, Cronbach alpha = 0.857), Negative Affect (6 items, Cronbach alpha = 0.946), and Habit/Addictive (5 items, Cronbach alpha = 0.800) [50].

#### Partner Interaction Questionnaire

Supportive behaviors from a spouse/partner have been shown to predict successful quitting [51,52], and negative behaviors predict relapse [53,54]. The Partner Interaction Questionnaire (PIQ) [53] is the most commonly used measure of spouse/partner support related to cessation. We administered a modified version of the PIQ that measures the receipt of specific behaviors from the person who follows the participant’s efforts to quit smoking most closely, not just a spouse/partner [55,56]. The modified version assessed how frequently the participant’s support person exhibited three positive and three negative behaviors [46], with responses of never (0), almost never (1), sometimes (2), fairly often (3), and very often (4). The three positive items were “express pleasure at your efforts to quit,” “congratulate you for your decision to quit smoking,” and “express confidence in your ability to quit/remain quit.” The three negative items were “mention being bothered by smoke,” “ask you to quit smoking,” and “criticize your smoking.” Cronbach alpha coefficients were 0.92 for the 3-item positive subscale and 0.84 for the 3-item negative subscale.

#### Perceived Stress Scale

Stress has been implicated in problems quitting smoking and in relapse [57]. The 4-item Perceived Stress Scale (PSS) [58] assessed the degree to which participants found their lives to be unpredictable and uncontrollable during the past month. Response options were never (0), almost never (1), sometimes (2), fairly often (3), and very often (4). Cronbach alpha reliability coefficients range from 0.60 to 0.72 [58,59]. Test-retest correlations range from 0.85 over 2 days in a college sample to 0.55 over 6 weeks in a smoking cessation sample [58].

#### Center for Epidemiological Studies Depression Scale

Symptoms of current depression were measured using the 10-item Center for Epidemiological Studies Depression Scale (CES-D) [60]. Scores on the CES-D have been positively associated with smoking prevalence and intensity and failure to quit in representative samples of US adults [61]. The CES-D is widely used in smoking cessation trials in the United States and abroad (eg, [62-67]). Each item is rated on a 4-point scale to indicate the frequency of occurrence during the past week. Response options were modified to less than one day (0), one to two days (1), three to four days (2), and five to seven days (3). Test-retest correlations range from 0.21 to 0.84, with an overall correlation of 0.71, at an average time interval of 22 days [60].

#### Alcohol Use

Alcohol use is a common barrier to cessation [68,69]. Participants were first asked if they drank any alcohol. Using items from the then current 2002 Behavioral Risk Factor Surveillance System [70], those who said yes were asked to indicate how many days per week on average they drank alcohol, how many drinks they typically had on a drinking day, and the maximum number of drinks they had on one occasion during the past month. In addition, we used a slightly modified version of a 2-item screener [71] to assess problems associated with alcohol use. The original questions asked about alcohol and drug use conjointly; our modification dropped the wording about other drugs so that questions read as follows: “In the last year, have you had more to drink than you meant to?” and “In the last year, have you felt you wanted or needed to cut down on your drinking?” These items have high specificity (80%-90%) to detect current alcohol problems.

#### Quit Methods

Participants indicated whether they had ever used various methods to quit smoking, including cold turkey, pamphlet or book, individual counseling, group counseling, nicotine patch, nicotine gum, nicotine nasal spray, nicotine lozenge, nicotine inhaler, Zyban (bupropion), switching to chewing tobacco or snuff, an Internet program (not including QuitNet), telephone counseling, acupuncture, hypnosis, or any other method.

#### Perceived Health Status and Medical History

Using the item from the Medical Outcomes Study 36-Item Short-Form Health Survey (SF-36), participants rated their current health status on a 5-point scale from 1 (excellent) to 5 (poor) [72]. Participants were also asked if they had ever had a smoking-related illness (yes/no).

#### Income

Income is considered a sensitive question that some participants may not be comfortable answering. We examined its reliability to determine if the greater anonymity of the Internet would result in different responses than telephone administration. Total household income during the past year was assessed with eight response options: less than US $10,000, $10,000-20,000, $20,000-30,000, $30,000-40,000, $40,000-50,000, $50,000-75,000, $75,000-100,000, and $100,000 or more.

### Statistical Analysis

The first set of analyses documents the recruitment process and describes the recruited sample, including a comparison of participation rates between the original study and the present study. To examine the generalizability of the final sample, we characterized survey participants on a range of demographic, smoking, and psychosocial variables. Frequency tables are used to summarize the categorical data, and both parametric and nonparametric tests are employed to determine the statistical significance levels.

The test-retest reliability of measures across modes of survey administration (Internet versus telephone) was examined by race/ethnicity and income. Specifically, we conducted stratified analyses that compared and contrasted (1) non-Hispanic White participants versus racial/ethnic minorities and (2) low-income versus high-income participants. The group of racial/ethnic minorities is comprised of participants who reported their race as African American, Asian, Native Hawaiian / Other Pacific Islander, or American Indian / Alaska Native, or their ethnicity as Hispanic. Based on a naturally occurring median split, the binary income variable was created with low income representing US $40,000 or less (49.2%) and high income representing more than US $40,000 (50.8%). We considered using educational level as a stratification variable instead of income but decided against it. Since only 21% of our subjects had a high school degree or less, the uneven sample size would have resulted in low power for testing differences between Internet- and telephone-administered measures among subjects with lower educational level, as well as imprecise estimates of the corresponding reliability coefficients.

In Table 1, the test-retest reliability of all continuous variables is examined across survey methods using the intraclass correlation coefficient (ICC), calculated according to formula ICC(3,1) of Shrout and Fleiss [73]. This version of the ICC measures the correlation between a single rating on a continuous measure using the Internet survey, with a single rating of the same measure obtained over the telephone, when Internet and telephone are the only channels of interest for administering the survey (fixed rater scenario). In large samples, the ICC has an F distribution that can be used to derive asymptotic 95% confidence interval (95% CI) estimates. Test-retest reliability above 80% is usually sought in method comparisons, with 70% considered an acceptable value.

 Since reliability measures are based upon mean-centered versions of the variables of interest, they are insensitive to participants’ tendencies to provide consistently higher responses on one survey instrument than another. Therefore, examination of test-retest reliability for these two survey methods was supplemented by *t* tests aimed at detecting the presence of any systematic bias as manifested by location differences between the Internet and telephone surveys. We report the results of these *t* tests below, but these data have been omitted from Table 1 due to space limitations (the complete set of tables is available upon request from the corresponding author). To allow for the presence of outliers in the data, robust location tests based on the Wilcoxon statistic were also carried out. Additionally, effect size measures based on standardized mean differences were estimated for each stratum, allowing us to distinguish clinically significant from merely statistically significant results. With approximately 160 subjects per stratum of interest, this study was designed to ensure detectability with at least 80% power at the 5% significance level of within-stratum location differences corresponding to a “small” effect size (delta = 0.20), when the within-subject correlation in the responses across the Internet and telephone surveys is no lower than 0.60.

In Tables 2 and 3, we examine differences in the test-retest reliability of binary and ordinal variables across the four strata. Although not presented due to space limitations, the prevalence of binary and ordinal variables was calculated for both Internet- and telephone-administered measures. Prevalence differences between the paired binary indicators contributed by each study subject were tested using the McNemar test of marginal homogeneity, as implemented in PROC FREQ of SAS v8.2 [74]. This test is equivalent to checking whether any disagreements that occur between the two methods of administration are entirely random and, hence, equally likely to be resolved in favor of either. It is noteworthy that its power is driven entirely by the number of subjects with discordant reports (N_D_) rather than the total sample size. Effect sizes for the sign test have been defined by Cohen [75] as "small" for *g* = 0.05, "moderate" for *g* = 0.15, and "large" for *g* = 0.25, where *g* is the absolute difference from 50% in the proportion of discordant pairs that endorse the Internet over the interviewer-administered measure. Detectability with 80% power at the 5% significance level requires that N_D_ exceeds 140, 79, and 23, respectively. On this basis, "small" prevalence differences between Internet- and telephone-administered measures are detectable for all variables listed in Table 2, other than for the alcohol-related questions, for which only "moderate" differences can be detected.

In testing for prevalence differences between ordinal variables in Table 3, a latent variable model is assumed in which these variables can be construed as discretized versions on an underlying continuous variable. This model holds exactly for household income and appears quite reasonable for measuring health status. Because of skewness, the probit link associated with normal data in the latent scale was replaced by a log-log link for income and a complementary log-log link for health; both links can accommodate departures from symmetry and are related to the Gumbel distribution. In this setting, tests for prevalence differences between the Internet- and interviewer-administered measures translate into tests of location differences in the latent scale, implemented in PROC GENMOD of SAS v8.2 using generalized estimating equations, with a working exchangeable correlation matrix used to adjust for within-subject dependence in the paired ordinal measurements.

Shown in Tables 2 and 3 are the kappa coefficients [76], which measure the level of between-method agreement beyond that which can be ascribed to chance. Kappa coefficients have their range constrained by differences in prevalence between the dichotomous measures under investigation, and caution should be exercised in their interpretation when the associated sign test is significant [77]. In the absence of prevalence differences, standard cutoffs for measuring agreement have been established by Landis and Koch [78], which rate them as follows: 0.80-1.00 = almost perfect, 0.60-0.80 = substantial, 0.40-0.60 = moderate, 0.20-0.40 = fair, 0.00-0.20 = slight, and < 0.00 = poor. Confidence intervals for kappa coefficients have been calculated in Tables 2 and 3 using the profile variance method of Lee and Tu [79], which improves on the more common asymptotic normal approximation of Fleiss et al [80]. Extensions of kappa-type statistics to ordinal data have been proposed by Cohen [81] and require weights for the cells corresponding to partial agreement. Linearly decreasing weights of the form 1 *−* (*i − j*) / (*k −* 1) are employed, where *i* and *j* refer to the row and column scores and *k* is the number of categories. Health status has been rated on a 5-point scale, whereas household income is scored using the category midpoints for all categories other than the last one for which a sensitivity analysis was conducted by varying the midpoint from US $125,000 to $150,000. Finally, in Table 4 the internal consistency of several continuous scales is examined using Cronbach alpha coefficient [82], with the 95% CI obtained according to van Zyl et al [83].

## Results

### Recruitment Results and Sample Characteristics

**Figure 1:**
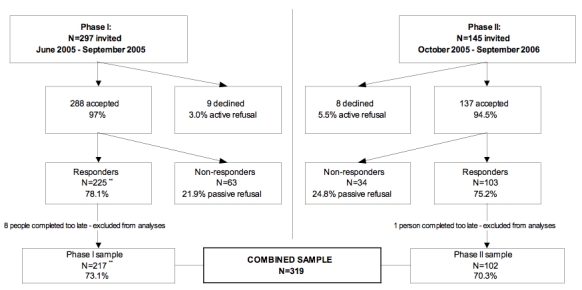
Flowchart of enrollment in phase I and phase II

Details about enrollment are provided in Figure 1. During phase I of recruitment (June-September 2005), 297 individuals were invited to participate: 288 accepted (97%) and 217 (73.1%) completed the online survey within 1 week of their telephone assessment. Four individuals completed the online survey after data were pulled for the original analyses presented in our earlier study [46]. Thus, the sample size and response rates vary slightly from our original manuscript. During phase II of recruitment (October 2005 to September 2006), 145 individuals were invited to participate: 137 accepted (94.5%) and 102 (70.3%) completed the online survey within 1 week of their telephone assessment. The final sample size was 319. With regard to race/ethnicity, 52.4% were non-Hispanic White, 22.9% Black, 11.6% Hispanic, 7.8% Asian, 4.4% American Indian / Alaska Native, and 1% Native Hawaiian / Other Pacific Islander. About half (49.4%) of participants reported an annual household income of US $40,000 or less and 25.7% had a high school degree or less. The majority were women (61.4%), the average age of participants was 35.23 years (SD = 10.9; range 18-78), and participants smoked an average of 17.9 cigarettes per day (SD = 9.4; range 5-60).

There were no significant differences in participation rates between phase I and phase II of the study. The “active refusal rate” (ie, those who declined the initial invitation to participate) was 3.0% in phase I and 5.5% in phase II (*χ* ^2^_1_ = 1.6, *P* = .20). The “passive refusal rate” (ie, those who accepted the invitation to participate but did not complete the online survey) was 21.9% in phase I and 24.8% in phase II (*χ* ^2^_1_ = 0.4, *P* = .49).

### Means and Prevalence Data

For variables with negligible missingness (Smoking Temptations, PIQ, PSS, CES-D) the stratum-specific sample sizes were non-Hispanic Whites = 167, racial/ethnic minorities = 151, high income = 163, and low income = 151; for these variables, the study had at least 80% power at the 5% level of significance to detect stratum-specific effect sizes of delta = 0.22-0.23. For the alcohol variables, for which missingness rates were higher, the corresponding sample sizes were non-Hispanic Whites = 124, racial/ethnic minorities = 199, high income = 123, and low income = 99; for these variables the minimum detectable effect size rose to delta = 0.25-0.28. According to Cohen's [75] nomenclature, these are "small" effect sizes which, while likely to be statistically significant in our study, may be of less practical import than "moderate" effect sizes in the delta = 0.50 range.

As shown in Table 1, there was little systematic bias between the two survey methods as indicated by strong ICCs across all continuous variables. In examining mean differences, the only variable showing differences of “moderate” effect size between the Internet- and telephone-administered questionnaires is the Negative Affect subscale of Smoking Temptations, with the mean of the interviewer-administered measures 0.42-0.54 standard units higher than higher than the mean of the Internet-administered version across strata (all *P* values < .001). As a result, the total score of the Smoking Temptations scale shows an overall mean difference in the “small to moderate” range, with the two sample means 0.29-0.44 standard units apart (all *P* values < .001). Despite statistically significant differences for variables such as the PIQ total score and the CES-D measured among non-Hispanic Whites, the observed effect sizes were “small,” a result of the ample power our sample size affords for detecting within-subject differences in continuous outcomes.

**Table 1:** Internet–telephone reliabilities of continuous variables by race/ethnicity and income

	Racial/Ethnic Minority		Non-Hispanic White		Low Income		High Income	
	ICC	95% CI	ICC	95% CI	ICC	95% CI	ICC	95% CI
**Smoking Temptations (total)**	0.73	0.65-0.80	0.67	0.58-0.75	0.67	0.57-0.75	0.73	0.65-0.79
Positive Affect or Social Situations	0.70	0.61-0.77	0.65	0.55-0.73	0.66	0.56-0.74	0.69	0.59-0.76
Negative Affect Situations	0.67	0.57-0.75	0.66	0.56-0.74	0.69	0.60-0.77	0.63	0.54-0.72
Habitual or Craving Situations	0.69	0.60-0.77	0.70	0.61-0.77	0.70	0.62-0.78	0.69	0.60-0.76
**PIQ (total)**	0.88	0.84-0.91	0.90	0.87-0.93	0.89	0.85-0.92	0.90	0.87-0.93
Positive	0.75	0.67-0.81	0.85	0.80-0.88	0.75	0.68-0.82	0.85	0.80-0.89
Negative	0.87	0.83-0.91	0.91	0.88-0.94	0.89	0.85-0.92	0.91	0.87-0.93
**PSS**	0.77	0.70-0.83	0.74	0.66-0.80	0.79	0.73-0.85	0.71	0.62-0.78
**CES-D**	0.81	0.75-0.86	0.78	0.71-0.83	0.79	0.72-0.84	0.79	0.72-0.84
**Alcohol Use**								
Number of drinking days per week	0.94	0.91-0.96	0.95	0.93-0.96	0.96	0.93-0.97	0.93	0.90-0.95
Number of drinks on a typical day	0.91	0.87-0.94	0.83	0.76-0.88	0.93	0.89-0.95	0.77	0.69-0.83
Max number of drinks on a single occasion	0.92	0.87-0.95	0.93	0.91-0.95	0.97	0.95-0.98	0.91	0.87-0.93

Of the binary variables listed in Table 2, only two variables showed statistically significant differences in prevalence between the two survey methods. Across strata, the prevalence of self- reported smoking-related illness was 7%-13% higher when assessed over the phone (all *P* values < .02). Among non-Hispanic White and low-income participants, the use of pamphlets or booklets as quit aids was 6%-7% higher when assessed over the Internet (non-Hispanic White: 24.5% vs 17.9%, *P* = .02; low income: 25.2% vs 19.4%, *P* < .02). It should be noted that the post hoc power of the McNemar test in the present study is quite low for all but “large” effect sizes due to the small number of discordant pairs (N_D_ < 26 throughout). The ordinal variables listed in Table 2 (income, health status) showed no significant differences in prevalence under a latent variable model (all *P* values > .09).

### Test-Retest Reliability and Internal Consistency Results

As seen in Table 1, test-retest reliability across modes of survey administration exceeded the minimal threshold of 70% for the majority of measures across strata. Reliabilities were very high (above 90%) for the alcohol use measures, with the exception of the number of drinks per typical day, for which reliabilities were lower for non-Hispanic Whites (0.83) and high-income subjects (0.77). For the PIQ, reliability was around 90% for both the total score and Negative Affect subscale, but dropped to 75% for the Positive Affect / Social subscale among racial/ethnic minority and low-income respondents. Reliability was moderately strong (in the 78%-81% range) for the CES-D and acceptable (in the 71%-79% range) for PSS. Results were least satisfactory for the individual subscales of the Smoking Temptations scale, none of which exceeded the 70% reliability threshold. Still, the overall scale (total score) was more reliable as would be expected from a composite of three correlated subscales, with its ICC exceeding the 70% threshold among racial/ethnic minority and high-income respondents.

In Table 2, almost perfect agreement between the two survey methods (kappa in the range of 0.80-1.00) was obtained across strata for 7 of the 15 binary variables assessing prior use of quit methods: nicotine patch, nicotine gum, nicotine inhaler, Zyban (bupropion), switching to chewing tobacco or snuff, acupuncture, and hypnosis. Use of the nicotine lozenge also showed near perfect agreement across all strata with the exception of racial/ethnic minority respondents, for which substantial agreement was obtained (kappa = 0.68). Three quit methods showed substantial degrees of agreement across all strata (kappa in the range of 0.60-0.80): use of pamphlet or booklet, group counseling, and telephone counseling. At least moderate agreement was obtained across strata for quitting cold turkey (kappa in the range of 0.56-0.71) and individual counseling (kappa in the range of 0.40-0.60). Agreement was poor to fair for Internet use (kappa in the range of 0.18-0.49). Reported use of nicotine spray as a quit method (which was infrequent among all respondents) showed poor agreement across surveys for racial/ethnic minority and low-income subjects, but moderate agreement among non-Hispanic White and high-income respondents. Although report of ever having a smoking-related illness showed substantial degrees of agreement across all respondents (kappa in the range of 0.65-0.71), this is a variable for which use of the kappa statistic may be inappropriate due to previously reported prevalence differences between the two survey methods [46]. As for the alcohol measures, all of them showed substantial to near perfect agreement across all four strata (all kappa values exceed 0.70).

**Table 2:** Internet–telephone reliabilities of binary variables by race/ethnicity and income

	Racial/Ethnic Minority		Non-Hispanic White		Low Income^*^		High Income^*^	
	Kappa^†^	95% CI	Kappa^†^	95% CI	Kappa^†^	95% CI	Kappa^†^	95% CI
**Quit Methods (ever used)**								
Cold turkey	0.69	0.52-0.86	0.58	0.42-0.73	0.71	0.55-0.88	0.56	0.40-0.72
Pamphlet or booklet	0.68^‡^	0.54-0.83	0.70	0.58-0.83	0.72^‡^	0.59-0.85	0.67	0.54-0.81
Individual counseling	0.57	0.31-0.84	0.48	0.13-0.84	0.61	0.34-0.87	0.44	0.09-0.78
Group counseling	0.69	0.51-0.88	0.80	0.63-0.97	0.68	0.48-0.87	0.81	0.65-0.97
Nicotine patch	0.96	0.91-1.00	0.92	0.85-0.98	0.95	0.90-1.00	0.93	0.87-0.98
Nicotine gum	0.91	0.84-0.98	0.92	0.86-0.98	0.90	0.82-0.97	0.93	0.88-0.99
Nicotine spray	−0.01	−0.02 to 0.00	0.66	0.05-1.00	−0.01	−0.02 to 0.00	0.49	−0.11 to 1.00
Nicotine lozenge	0.68	0.44-0.92	0.91	0.81-1.00	0.80	0.63-0.97	0.85	0.71-0.99
Nicotine inhaler	0.88	0.72-1.00	0.85	0.68-1.00	0.89	0.75-1.00	0.84	0.69-0.99
Zyban (bupropion)	0.93	0.86-1.00	0.94	0.88-0.99	0.90	0.83-0.98	0.96	0.91-1.00
Switch to chewing tobacco or snuff	0.85	0.68-1.00	0.86	0.73-1.00	0.89	0.6-1.00	0.82	0.65-0.99
Internet program	0.18	−0.09 to 0.44	0.49	0.23-0.75	0.37	0.11-0.63	0.32	0.02-0.62
Telephone counseling	0.88	0.72-1.00	0.65	0.37-0.94	0.74	0.53-0.96	0.83	0.59-1.00
Acupuncture	0.85	0.65-1.00	1.00	1.00-1.00	0.93	0.79-1.00	0.95	0.85-1.00
Hypnosis	0.96	0.88-1.00	0.96	0.90-1.00	0.91	0.81-1.00	1.00	1.00-1.00
**Smoking-Related Illness**								
Ever had smoking-related illness?	0.65^‡^	0.54-0.77	0.71^‡^	0.61-0.82	0.67^‡^	0.55-0.78	0.70^‡^	0.60-0.81
**Alcohol Use**								
Do you drink alcohol?	0.91	0.84-0.98	0.90	0.82-0.98	0.90	0.83-0.97	0.91	0.84-0.99
More to drink than meant to	0.75	0.63-0.88	0.91	0.84-0.99	0.84	0.73-0.95	0.84	0.74-0.94
Wanted/needed to cut down	0.77	0.63-0.90	0.84	0.72-0.96	0.72	0.57-0.88	0.88	0.78-0.98

^*^Income scored at category midpoint; US $125,000 used for last category.

^†^Weighted kappa using absolute difference between category scores to define a distance measure.

^‡^McNemar test for prevalence differences is significant.

In Table 3 we find almost perfect agreement for the income measure (weighted kappa values > 0.84) and substantial agreement for health status (weighted kappa values > 0.72). Results for the income measure were not dependent on whether the midpoint of the highest income category used to construct the weights was changed from US $125,000 to $150,000. Due to the informativeness of ordinal (as opposed to binary) measures, the confidence intervals are narrower, which indicates improved precision in the estimates.

**Table 3:** Internet–telephone reliabilities of ordinal variables by race/ethnicity and income

	Racial/Ethnic Minority		Non-Hispanic White		Low Income^*^		High Income^*^	
	Kappa^†^	95% CI	Kappa^†^	95% CI	Kappa^†^	95% CI	Kappa^†^	95% CI
Income^†^	0.87	0.82-0.93	0.94	0.90-0.97	0.84	0.78-0.91	0.92	0.88-0.97
Health status	0.73	0.66-0.81	0.72	0.64-0.81	0.72	0.64-0.80	0.74	0.66-0.83

^*^Income scored at category midpoint; US $125,000 used for last category.

^†^Weighted kappa using absolute difference between category scores to define a distance measure.

Finally, Table 4 reports the internal consistency of four scales of interest (total score only) and contrasts it across survey methods. For all four scales, the Internet-administered versions consistently have higher internal consistency than the interviewer-administered ones. Across all four strata, Cronbach alpha coefficients approach or exceed 80% for CES-D under both methods, are in the 70%-80% range for the PIQ and PSS, and only fall below 70% for the Smoking Temptations scale. Cross-survey comparisons show no statistically significant differences for PIQ, PSS, and Smoking Temptations, but are significant at the 5% level across all strata for the CES-D scale. Although not reported in Table 4 due to space considerations, we also examined internal consistency of each of the three subscales of the Smoking Temptations Questionnaire within each of the four strata. The Negative Affect subscale maintained acceptable internal consistency levels across all four strata of interest, in the range of 77%-85% for Internet administration and 76%-78% for telephone administration. This was not the case for the Positive Affect / Social and Habit/Addictive scales, for which internal consistency levels never exceeded 60% in any of the four strata under both methods of administration. Full tables are available from the corresponding author.

**Table 4:** Internal consistency of measurement scales: Internet–telephone comparisons stratified by race/ethnicity and income

		Internet Administered		Telephone Administered	
	No. of Items^*^	Alpha^†^	95% CI	Alpha^†^	95% CI
**Smoking Temptations**					
Racial/ethnic minority	9	0.70	0.61-0.76	0.61	0.51-0.70
Non-Hispanic White	9	0.65	0.56-0.72	0.57	0.45-0.65
Low income	9	0.66	0.57-0.73	0.58	0.47-0.67
High income	9	0.68	0.60-0.75	0.60	0.50-0.69
**PIQ**					
Racial/ethnic minority	6	0.79	0.73-0.84	0.74	0.67-0.80
Non-Hispanic White	6	0.80	0.74-0.84	0.76	0.69-0.81
Low income	6	0.81	0.76-0.85	0.78	0.72-0.83
High income	6	0.79	0.73-0.83	0.72	0.65-0.78
**PSS**					
Racial/ethnic minority	4	0.75	0.68-0.81	0.75	0.68-0.81
Non-Hispanic White	4	0.77	0.71-0.82	0.71	0.62-0.77
Low income	4	0.75	0.67-0.80	0.72	0.63-0.78
High income	4	0.77	0.70-0.82	0.74	0.67-0.80
**CES-D**					
Racial/ethnic minority	10	0.86	0.82-0.89	0.82	0.77-0.86
Non-Hispanic White	10	0.86	0.82-0.89	0.79	0.73-0.83
Low income	10	0.85	0.81-0.88	0.79	0.73-0.83
High income	10	0.85	0.81-0.88	0.82	0.77-0.86

^*^Number of items in measurement scale.

^†^Cronbach alpha based on total score of unstandardized items for each scale.

## Discussion

This study demonstrated that the psychometric properties of a broad range of measures commonly used in smoking cessation clinical trials are not different when administered via the Internet to racial/ethnic minority or low-income participants. Few studies to date have explicitly examined race/ethnicity and income with sufficient sample size and power to determine the degree of consistency between Internet- and telephone-administered questions, and none have examined these questions for cessation constructs. Therefore, these results provide new and largely reassuring information about measurement and method variance across two modes of administration in samples of participants who are of increasing importance to researchers involved in tobacco use behavior and cessation intervention research. Given the high smoking prevalence rates among racial/ethnic minority and low-income individuals, it is important to be able to reach and intervene in these target groups and to know that important data about key variables such as mediators, moderators, covariates, and outcomes can be collected using efficient modalities such as the Internet.

While the majority of measures were consistent across modes of administration, there were several statistically significant differences in means and prevalence. In general, these differences have minimal clinical significance as they were small in magnitude; however, such differences highlight the importance of pilot testing items with the target population to ensure adequate comprehension of questions as well as response formats. Items that require clarification by telephone or that yield different means or prevalence when administered via the Internet may require more detailed instructions or specific illustrative examples to assist research participants. The Internet is more similar to a paper-and-pencil test than an interview during which prompts and clarifications can be made. Equivalent forms of questions need to be tested to ensure all items and scales are consistent across modes of delivery whenever possible. Detecting differences can help improve the reliability, validity, and equivalency of measures across modalities. Empirical data of the kind collected in this study can provide valuable information to researchers about possible sources of error variance or systematic measurement bias.

The majority of the test-retest reliability coefficients fell above the minimum threshold of 0.70, indicating substantial to strong agreement between survey methods. Two exceptions noted were in the quit methods measure in items that assessed use of nicotine spray and Internet cessation websites in previous quit attempts. In general, these two findings should be interpreted with caution given that overall prevalence of both quit methods was very low in both the phone and Internet surveys (< 10%) and that both point and interval estimates of kappa are extremely sensitive to small changes in cell counts. However, we also know from analyses of follow-up data for the parent study that some participants continued looking for cessation assistance on the Internet following randomization. When reporting on use of smoking cessation websites in the Internet survey, participants may have included their use of cessation websites following the baseline telephone assessment. The take-home message here is that it is critical to examine the time frame referenced in a reliability study to ensure that the wording of questions does not artificially inflate or deflate the concordance of responses. Participants were asked to indicate whether they had “ever used” a variety of quit methods. It is reasonable to consider that participants in the smoking cessation parent trial began trying various methods of quitting immediately following enrollment and referenced those methods in the Internet survey (administered following randomization) but not in the baseline telephone survey.

Internal consistency across items was good for all scales examined, with the exception of the Smoking Temptations scale. Across strata, Cronbach alpha coefficients did not exceed the threshold of 0.70 for either the total scale score or any of the subscales. These findings are consistent with our previously published study [46] and with work by Ward (personal communication, RM Ward, October 2007) in which Cronbach alpha coefficients were as follows: Negative Affect = 0.765, Habit/Addictive = 0.579, and Positive Affect / Social = 0.573. Given the poor performance of the Habit/Addictive and Positive Affect / Social subscales, it is not surprising that the internal consistency of the overall Smoking Temptations scale failed to exceed the 70% threshold across strata and mode of administration in the present study. Given these findings, further refinement of the Short-Form of this measure is called for, especially since the availability of psychometrically sound, brief assessment instruments is critical to minimize response burden in Web-based smoking cessation research trials.

Results should be considered in the context of several limitations. First, it is possible that the higher internal consistency seen in Internet-administered measures was due to learning effects since Internet measures were always administered several days after the telephone interview. Counterbalancing the order of administration would address this limitation and should be considered in future studies. Second, it is possible that the internal consistency may have been artificially inflated due to memory effects associated with the relatively short (ie, 2-7 days) time frame between measurement points. There is often a 2-4 week gap between repeat administrations of the same scales for test-retest reliability studies. Given the dynamic nature of many of the constructs we assessed—especially in the context of a cessation trial—this shorter time frame was necessary so as not to artificially deflate internal consistency due to expected changes in knowledge, attitudes, and beliefs. Third, some may question our use of a cutoff of US $40,000 for our “low income” stratum. The median household income in the United States 2006 was US $48,201 [84], meaning that half the US population fell below this threshold. In addition, Internet use is more common in households with higher levels of income (93% for ≥ US $75,000 vs 49% for < US $30,000 [14]). Therefore, we believe that US $40,000 or less is a reasonable cutoff for lower income Internet users. Finally, our use of race/ethnicity as a categorization variable was to explore in a preliminary fashion whether there are differences by culture or context in the psychometric properties of Internet-administered measures. A limitation of this approach is that the group of racial/ethnic minority participants is likely still quite heterogeneous with regard to race, ethnicity, and other variables that may influence survey response patterns. Future studies should move beyond race and ethnicity to investigate the specific factors that may link race/ethnicity to measurement issues such as health literacy, technology access and familiarity, and other cultural factors.

In conclusion, the present study replicated findings from an earlier study demonstrating adequate internal consistency and test-retest reliability of a broad range of measures commonly used in smoking cessation clinical trials. In addition, this study extended these findings by examining measures among racial/ethnic minorities and individuals with lower levels of household income. This knowledge adds to the confidence of conducting Web-based research and strengthens the scientific rigor of collecting information via the Internet on racial/ethnic minority and low-income subgroups. This study also revealed a few areas where measurement scales did not perform as well as expected. These findings underscore the importance of explicitly testing consistency among subgroups with sufficient statistical power in order to test empirically the equivalence of measures and to identify measures that require more work to improve their performance in specific subgroups.
